# Drug pricing models, no ‘one-size-fits-all’ approach: a systematic review and critical evaluation of pricing models in an evolving pharmaceutical landscape

**DOI:** 10.1007/s10198-024-01731-w

**Published:** 2024-11-04

**Authors:** Evert A. Manders, Sibren van den Berg, Saco J. de Visser, Carla E. M. Hollak

**Affiliations:** 1 Medicine for Society, Platform at Amsterdam University Medical Center, Amsterdam, The Netherlands; 2 Department of Endocrinology and Metabolism, Amsterdam University Medical Center, Meibergdreef 9, Amsterdam, The Netherlands; 3 Centre for Future Affordable & Sustainable Therapy Development (FAST), The Hague, The Netherlands

**Keywords:** Drug pricing, Drug costs, Drug pricing models, Affordability, Innovation, I18, I19

## Abstract

Access to new medicines is crucial for patients but increasingly sparks discussion due to high prices. Simultaneously, the growing emphasis on specialized products and uncertainty surrounding the long-term effectiveness of new drug classes brought to the market underscore the need for innovative pricing approaches. A systematic literature review of pharmaceutical pricing models, accompanied by a critical appraisal, was conducted to offer insights contributing to novel approaches balancing sustainable pharmaceutical innovation with affordability and accessibility for patients. Six different pricing models are identified: value based pricing, basic cost-based pricing, and four more comprehensive pricing models incorporating numerous elements: the cancer-drug-pricing model, AIM model, (Nuijtens) discounted cash flow, and the real-option rate of return method. Although there are many similarities among the models, each has unique assumptions for implementation. For instance, all models except for the standard incremental cost-effectiveness ratio and basic cost-based pricing consider the number of eligible patients and the remaining patent period. Only the AIM model and the Nuijtens discounted cash flow model use lump sums. Both the latter and the real-option rate of return method explicitly include the cost of capital as a major cost-based component. Recognizing the diverse applications of each model highlights the need for more differential and dynamic pricing tailored to the characteristics and therapeutic areas of each drug. Additionally, the study underscores the importance of cost transparency in achieving this goal. Consequently, these findings can help stakeholders develop sustainable and affordable drug pricing mechanisms that address the complexities of the ever-changing pharmaceutical landscape.

## Introduction

Pharmaceutical expenses constitute a substantial portion of overall healthcare spending [1, 2] and are considered to negatively impact healthcare systems’ ability to provide widespread access to medicines [3]. Consequently, the drug prices set by pharmaceutical companies are a heavily debated topic with considerable criticism of industry profits and business models [4–6]. The argument in defence posits that these costs are inevitable for innovation given the drug development process, which is characterized by a high failure rate (90% from the start of clinical development to marketing authorization), extensive timelines (10–15 years), and substantial required investments [7–9]. Critics however, contend that the majority of industry spending would be allocated to marketing and sales rather than research and development (R&D) [4]. Moreover, high profit margins would be unwarranted since newly introduced medications, often priced higher than existing counterparts, would sometimes offer minimal additional clinical advantages [10–13]. However, truly innovative medicines can be of extremely high value to patient groups facing an unmet need [14].

This discussion on drug prices aligns with important trends in the pharmaceutical sector. *Firstly*, there has been a significant shift of R&D away from large pharmaceutical companies towards small businesses and academia. Major firms increasingly rely on mergers and acquisitions (M&A) for portfolio enrichment, obtaining products from small startups rather than pursuing in-house development [15, 16]. Concurrently, an increasing number of drugs originate from academia and reach the market through public- private collaboration [17, 18]. The use of public funding for drug innovations raises concerns; although pharmaceutical companies make significant investments and play a major role in product development, it can be argued that private entities benefit from public investments, while the risks are socialized [19]. This would create a scenario in which governments effectively pay for medicines both through research and development and by bearing high prices upon approval [20].

*Secondly*, there is a growing emphasis on innovations targeting specific populations, such as patients with rare diseases, or with the development of precision medicine. Traditionally, pharmaceutical companies pursued broad-application drugs for widespread use, yielding substantial earnings at once (referred to as ‘blockbusters’). However, advancements in the scientific understanding of disease pathways have prompted a shift toward precision medicine, including gene therapies, somatic cell therapies, and tissue-engineered products tailored for specific patient populations (‘niche busters’) [21–23]. For these products, there is often uncertainty about long-term effectiveness and safety outcomes, raising questions about how prices should be determined, given that in many countries clinical effectiveness and the ‘value’ a drug brings are focal points in drug price negotiations [24–26].

These developments within the industry, coupled with the uncertainty surrounding new drug classes entering the market and ongoing discussions on pharmaceutical prices, make it evident that it is time to critically examine how the price of a medicine is determined. It is therefore imperative to explore pricing methods that balance sustainable pharmaceutical innovation while ensuring affordability and accessibility for patients, a concern already recognized by the European Commission [27].

Currently, many countries rely on health technology assessments (HTAs) to evaluate the clinical effectiveness and economic impact of new pharmaceuticals, the outcomes of which play a pivotal role in price negotiations with pharmaceutical companies [28, 29]. Additionally, governments seek to control medicine prices through methods such as establishing maximum prices or determining prices based on international comparisons and those of similar existing products, respectively called external reference pricing and internal reference pricing [3, 29]. However, much is still unknown about the precise models and calculations which can be used to determine a potential price for a medication, while the successful implementation of alternative drug pricing methods first requires a thorough understanding of these models, considering their strengths, weaknesses, and applicability.

This paper aims to take an initial step in this direction and seeks to enhance the understanding of existing publicly available pharmaceutical pricing models through a systematic review. Consequently, the research objective is to map existing drug pricing models, critically assess the merits and limitations and relevance in various contexts, and evaluate them in light of ongoing discussions about drug prices and future affordable and sustainable therapy development.

## Methodology

### Research approach & scope

To gain a comprehensive understanding of drug pricing models as well as to critically assess their merits and limitations, a systematic literature review [30, 31] was conducted between September 2023 and 31 December 2023. Herein, the focus was exclusively on drug pricing models and their quantifiable components, intentionally omitting policy aspects of drug pricing and accessibility, such as government regulations and procurement approaches. Due to their variability across EU member states and their distinct influence on drug pricing, these aspects are beyond the scope of this review.

### Search strategy

A comprehensive search strategy was employed to explore the existing literature on drug pricing models. Both Web of Science and Google Scholar, the latter to allow inclusion of non-scientific publications, served as databases to locate articles, reports, and studies related to drug pricing models. The search string can be found in Table 1.

**Table 1:** Overview of the search strategy

*Database:*	*Search string:*
Web of Science	("Drug pricing model" OR "Drug pricing approach" OR "Drug price calculation" OR "Drug cost calculation" OR "Drug pricing schemes") OR (("Value-based pricing model" OR "Cost-based pricing model" OR "Performance-based pricing model") AND ("Drug" OR "Medicinal product"))
Google Scholar	("Drug pricing model" OR "Drug pricing approach" OR "Drug price calculation" OR "Drug cost calculation" OR "Drug pricing schemes") OR (("Value-based pricing model" OR "Cost-based pricing model" OR "Performance-based pricing model") AND ("Drug" OR "Medicinal product"))

The first part of the query included a diverse range of terms associated with drug pricing models. The second component focused on specific types of pricing models, already described by the World Health Organisation (WHO) [3, 29]. The third component ensured the inclusion of only articles directly relevant to the research objectives. Backward snowballing (also known as reference mining) was employed to thoroughly review the existing body of literature and identify additional sources describing drug pricing models by incorporating and screening the references of included studies into the dataset. Furthermore, to gain a profound understanding of the identified pricing models, information on these models was supplemented with data from other sources and publications.

### Inclusion and exclusion criteria

In order to ensure that the selected articles are accessible, recent, and focus specifically on papers or books that contribute to the understanding of drug pricing models the following criteria were used:

- **Publication date:** Articles published and made available online between 1 January 2013 and 31 December 2023 are considered for inclusion.
- **Accessibility:** Articles without full-text access are excluded.
- **Language:** Only articles written in English and Dutch are included for analysis.
- **Publication type:** Papers or books are included, while editorials, posters, and conference abstracts are excluded due to their limited content
- **Relevance to drug pricing models:** The study must describe or introduce a drug pricing model or an approach to pricing drugs to be considered for inclusion.
- **Measurable numeric parameters:** Included articles must feature drug pricing models that, based on quantitative input variables and calculations can be used to determine the price of an individual drug

### Screening and selection process

The screening and selection process involved a two-stage approach. First, titles and abstracts of identified papers were screened using Rayyan, a web-based tool designed for systematic reviews [32], by two reviewers, EM and SB independently. The use of Rayyan facilitated a collaborative workflow by allowing multiple researchers to access and screen the same set of articles simultaneously. Any disagreements were resolved through discussion and consensus. In the second stage, full-text articles were evaluated, and once again, discrepancies were addressed through discussion and consensus.

### Dissection, analysis, and synthesis

A data extraction form was utilized for the systematic collection of relevant information from the selected studies (Appendix 1). The extracted data encompassed, among other details, publication information (e.g., authors, publication year), details about the model and its parameters, and key findings. Subsequently, the extracted data were synthesized to provide a structured overview of the existing pharmaceutical pricing models and their components through narrative analysis, a qualitative research method involving the systematic examination and interpretation of textual or narrative data to identify patterns, themes, and insights [33].

## Results

**Fig. 1:**
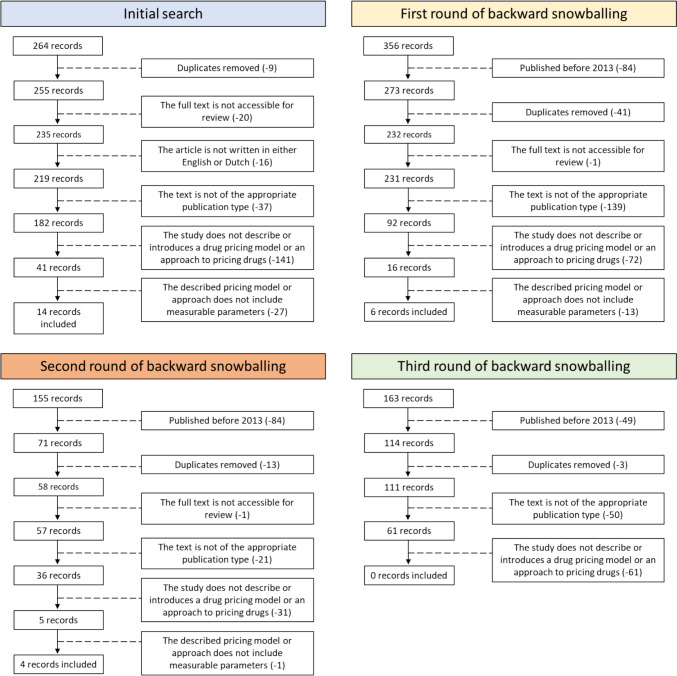
Overview of the screening and selection process

After removing duplicates and articles outside the time scope of this search, the applied search string yielded an initial set of 264 results. Following assessment and application of the pre-defined inclusion criteria, 14 studies were selected for further analysis. Through snowballing, 671 additional articles were screened, resulting in the inclusion of 10 more articles. In total, 935 articles underwent screening, and ultimately, 24 articles were included in the final analysis (Fig. 1).

Six distinct drug pricing models with measurable parameters could be retrieved from the 24 publications, since in some the same models were presented (Table 2).

**Table 2:** Overview of the included studies that emerged in the review and the model they describe

Identified model:	Included articles in review:
Value based pricing & the incremental cost-effectiveness ratio:	[34–38]
Basic cost-based (plus) pricing:	[39–45]
Cancer-drug pricing model:	[46, 47]
AIM model:	[48–50]
(Nuijtens) Discounted cashflow:	[35, 51–56]
Real-option rate of return pricing:	[57]

### Value based pricing & the incremental cost-effectiveness ratio

Value-based pricing (VBP) revolves around the idea that the price of a product or service should be aligned with its perceived or estimated value [58, 59]. In drug pricing, this involves establishing a price based on the health benefits a drug provides, defined as the sum of the best alternative price and the therapy’s differential value [34–37, 60]. The incremental cost-effectiveness ratio (ICER) is a specific metric that can be used within cost-effectiveness analysis (CEA) of a new drug, expressing the cost per unit of health benefit gained of one treatment over another [61, 62]. The primary metric often used is the cost per additional quality-adjusted life year (QALY) [60], as shown below:

$$ICER=\Delta Costs/\Delta QALYs$$

where,

- **∆Costs:** Represents the incremental costs associated with the new treatment compared to standard care
- **∆QALY:** Represents the incremental gain in quality-adjusted life years.

An alternative to this ICER is the incremental net monetary benefit, which considers how much decision-makers are willing to pay for each unit of benefit gained [35, 63, 64].

$$NMB = \Delta QALYs\times \lambda -\Delta Costs$$

where,

- **λ (lambda):** Represents the payer’s willingness to pay for each incremental QALY gained

Given that CEAs are a focal point in HTAs and pricing negotiations between health payers and pharmaceutical companies, VBP is incorporated to some degree in many nations [28]. For the operationalization of this, various nations utilize cost-effectiveness thresholds. In the UK, these thresholds range from £20,000 to £50,000 per QALY, while in the US, the range spans from $50,000 to $150,000 per QALY [34, 63]. The maximum price for a medication can be determined based on this threshold and the ICER. From a firm’s perspective, this price also represents the profit-maximizing price, considering the usage permitted by the payer at that price [37].

The core idea behind VBP is that medical innovations should be rewarded and priced based on the value they provide. Consequently, by linking rewards for innovation directly to provided benefits, VBP aims to incentivize pharmaceutical developers to prioritize projects with higher anticipated clinical returns [60]. However, as described in the introduction, accurately determining a product’s value can be challenging, especially for novel classes of drugs entering the market and precision medicine [24–26]. Nevertheless, evaluating the benefits of a drug is not impossible, as demonstrated by the evaluations conducted by the American Institute for Clinical and Economic Review and the British National Institute for Health and Care Excellence [65]. For this purpose, Markov models or simulations are often employed, which study systems undergoing transitions between different states over time [66].

### Basic cost-based (plus) pricing

Cost-based pricing is an approach that considers the costs involved in producing goods to determine the price.

While usually applied at the manufacturer level, this approach can also extend to setting prices for reimbursement by healthcare systems [3, 29]. To establish the reimbursed price, national authorities require actual cost data up to the point where the price can be set, and a mutually acceptable profit margin can be negotiated with the pharmaceutical company (known as cost-plus pricing) [3, 29]. Drawbacks of cost-based pricing include the challenge of obtaining actual information from manufacturers and its failure to provide incentives for innovation, as R&D costs would not be compensated, limiting its applicability to pricing generic medicines only. Due to the lack of an agreed framework among stakeholders for determining input costs, the WHO furthermore advises against using cost-based pricing as the primary policy for setting drug prices [3].

**Fig. 2:**
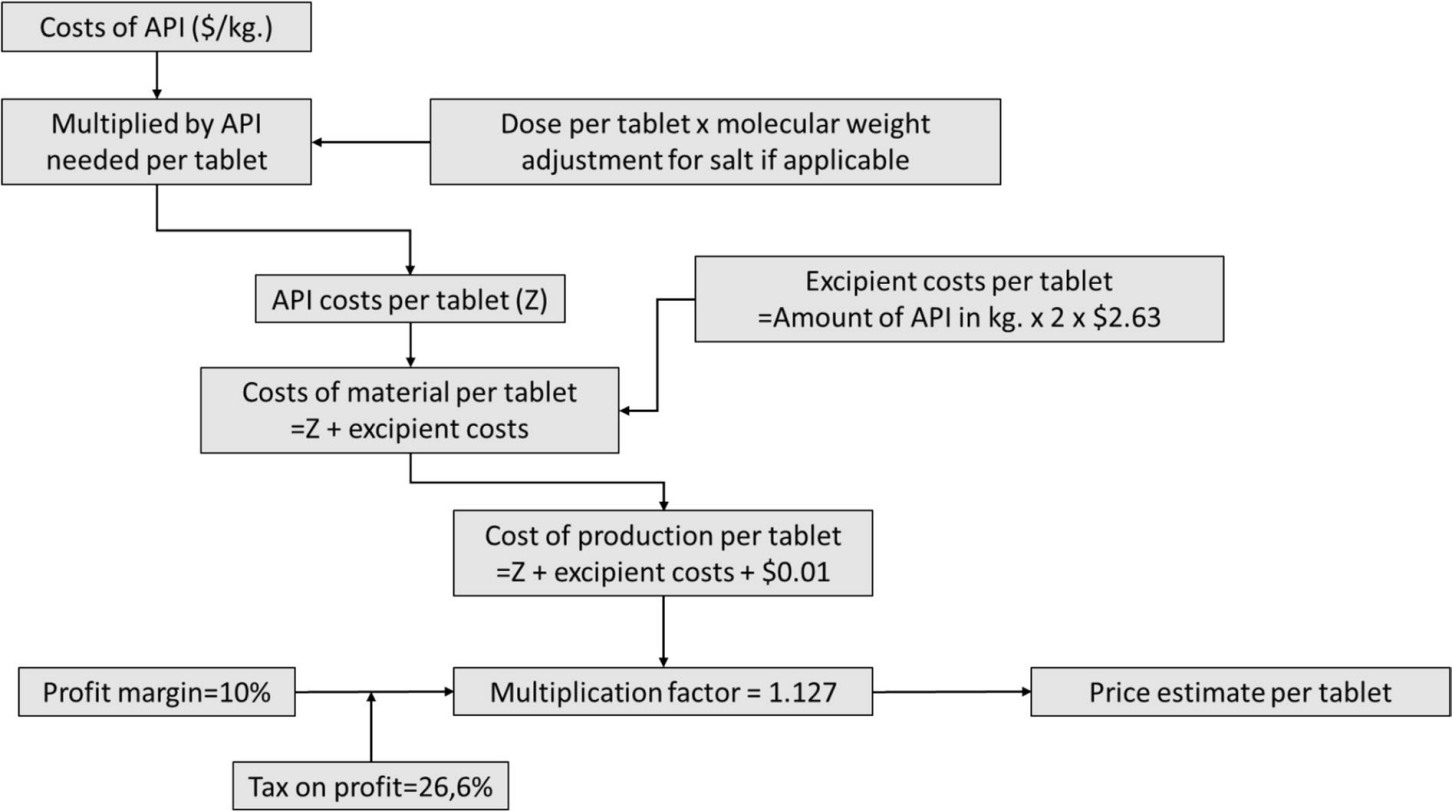
Framework for cost-based pricing, adapted from Hill et al. (2018) [39]

Efforts have been made to develop such a standardized framework, which has already been applied to generic medicines on the WHO Essential Medicines List, insulin analogues, treatments for tuberculosis, and cancer medicines [39–44]. This would involve examining the costs of the active pharmaceutical ingredient (API), the costs of excipients, and conversion costs (Fig. 2). Additionally, a small profit margin of 10% would be incorporated.

An alternative perspective to cost-based pricing has also been proposed, where R&D costs are considered [45]. In this approach, the capital costs required to develop a drug are adjusted based on the gross domestic product (GDP) share of the Organisation for Economic Co-operation and Development (OECD), and then divided by the patent period years. This yields the amount that needs to be recovered annually by the pharmaceutical company to break even, which would then be divided by the number of patients to arrive at a price per patient per year (Pppy). However, the proposed approach does not specify all the components included in capital costs, making the model difficult to apply in practice.

### Cancer-drug pricing model

Uyl-de Groot and Löwenberg (2018) have proposed a model which aims to determine a reasonable price for cancer drugs [46]. In this model, the price of a drug is determined based on multiple factors such as R&D costs, manufacturing, sales, marketing, and clinical benefits. Additionally, it considers the number of patent years remaining after registration and the number of eligible patients. While initially designed for oncology drugs, this model has already been applied to Libmeldy and Zolgensma, two non-oncology gene therapies [47]. The price is calculated as follows:

$$\text{Cos} t\,price\,of\,new\,drug\,treatment = ((\text{Cos} t\,of\,R\& D/number\,of\,patients \times years\,left\,of\,patent) + \cos t\,of\,drug) \times (1 + profit\,m\arg in)$$

The ‘drug cost’ parameter includes manufacturing, sales, and marketing expenses, with the latter projected to constitute 30% of the manufacturing cost. Profit margins are linked to expected clinical benefit, with higher percentages for greater benefit levels (20% for ‘marginal,’ 30% for ‘moderate,’ and 40% for ‘high’). In this model, clinical benefit for oncology products is determined using the ASCO Value in Cancer Care Framework or the ESMO Magnitude of Clinical Benefit Scale (ESMO-MCBS), where profit margins appear to have been chosen somewhat arbitrarily. To address the varying willingness to pay of drugs across countries, it is recommended to establish a maximum price at the national level. If the proposed pricing results in an ICER exceeding a specific threshold, the authors suggest that the drug might not provide sufficient value for the costs. Nonetheless, exceptions can be considered, taking into account factors such as disease severity, orphan disease status, the absence of alternatives, or limited budget impact. Additionally, the authors argue that prices should be periodically revisited and adjusted at defined intervals as new information on clinical benefits may emerge over time, and the approved indications of a drug may change. These developments could potentially impact the anticipated level of clinical benefit and the pool of eligible patients. Therefore, it is suggested to start with a fixed pricing period, such as three years.

Concerns have been raised about the proposed model, including its omission of opportunity costs and the ex-ante nature of investment decisions involving factors such as the cost of capital (CoC), time value of money, and risk [67]. In the previously mentioned application of this model for Zolgensma and Libmeldy, however, these aspects were taken into account, demonstrating the feasibility of including these components within the model [47]. Another critique is that patent holders could manipulate the ‘years remaining on the patent’ parameter to maximize financial gain, as patents are often extended or renewed based on additional research or changes in drug formulation, making estimates of remaining patent life unreliable [68].

### AIM model

The International Association of Mutual Benefit Societies (AIM) has developed a pricing model that includes the R&D expenses, the production costs, a (restricted) budget for sales, and an innovation bonus to incentivize the development of medicines offering added therapeutic value [49]. The first practical application of this model was to calculate the price for mexiletine, a repurposed drug prescribed for nondystrophic myotonia, demonstrating its real-world relevance [50]. The model calculates the average fair price using the following formula:

$$Average\,fair\,price = realR\& D\,\cos ts/number\,of\,patients + real\,production\,and\,overhead\,\cos ts + sales\,and\,medical\,\inf ormation + profit\,before\,tax + innovation\,bonus$$

Developed to provide incentives for R&D investments, the model aims to encourage companies to focus on developing self-originated new chemical entities instead of relying on M&A to acquire products for their portfolio. In line with this objective, the model assigns a lump sum R&D expenditure ranging up to €2.5 billion, with an initial €250 million allocated for each new drug, reflecting the minimum research investment required to bring a new drug to market. AIM outlines how companies can substantiate higher R&D expenses and request additional funding by providing evidence of their actual R&D costs. However, in order to prevent the inclusion of excessively expensive mergers and acquisitions under R&D expenditure, the maximum justifiable R&D costs are capped at €2.5 billion.

The model operates under specific assumptions. Firstly, it was developed for the European market, presuming that 35.85% of the total R&D costs are allocated to Europe, which corresponds to the financial share of the European market among all main developed markets. These costs are spread over the assumed treatment duration, which is ten years for chronic conditions. Additionally, it is expected that treatment is provided to 50% of the target population over a ten-year period (the average patent protection period after market authorization), and that the market is equally divided among competitors. Production costs include manufacturing expenses, production investments (e.g., factories, packaging), and distribution costs (e.g., warehousing, transport, taxes). For orphan drugs, these production costs are multiplied by 5 for limited production volume, while for high-prevalence diseases these may be manually limited to account for economies of scale. Sales expenses are capped at 20% of total R&D expenses, with a baseline profit of 8% of total costs to ensure a balanced allocation of funds between marketing and R&D. The model also introduces an innovation bonus, ranging from 5 to 40% of total costs, depending on the therapeutic value of the drug. For drug repurposing and the addition of new indications, the new price is determined as the weighted average of the price for each indication. In this calculation, a lump sum of 10% of the initial R&D costs is allocated for research and development expenses.

It should be noted that an important caution in this model is that it is ambiguous whether costs of failed trials and products that do not reach the market, as well as CoC, are included under the R&D expenses. In some literature, upon which AIM relies to derive its range of lump sums, this is indeed the case, although it is not explicitly stated in the model. This could be an explanation for the large range for R&D costs used in the model and the higher values within this spectrum. Moreover, the model does not provide lump sum estimates for production costs, which complicates its applicability, requiring transparency or self-made estimates.

### (Nuijtens) Discounted cash flow

The Discounted Cash Flow (DCF) method is a basic financial analysis tool used to determine the current value of (future) cash flows [69]. By factoring in the anticipated cash flows and the required CoC, the DCF method aims to provide a framework for evaluating a project’s profitability and its adherence to investors’ expectations. This versatile economic method can also be applied to value drugs in a pharmaceutical company’s portfolio [56, 70, 71]. The formula guiding this analysis is as follows:

$$NPV=C{F}_{1}/(1+r{)}^{1}+C{F}_{2}/(1+r{)}^{2}+...+C{F}_{n}/(1+r{)}^{n}$$

where,

- **NPV (Net Present Value):** The current value of future cash flows, indicating the worth of an investment or project in today’s terms.
- **CF (Free Cash Flow):** Expected cash flows at different time periods.
- **n:** The number of years into the future that the cash flows are expected.
- **r (Discount rate):** The rate used to discount future cash flows back to their present value.

The DCF method adjusts future cash flows to their present value using a discount rate, which typically includes the CoC and the expected returns that investors require. When the NPV equals zero, it indicates the breakeven point, covering the projects initial investment. A positive NPV indicates profitability, while a negative NPV signifies a loss.

It should be noted that the NPV calculation is a general economic tool where a high level of confidence in the input parameters is required to arrive at a reliable and meaningful assessment (i.e. “garbage in, garbage out”) [72]. Additionally, numerous factors can be incorporated into the discount rate, making the calculation as complex as desired. Consequently, there is no uniform approach to applying this DCF valuation, as evidenced by the various applications of this method to value and price medical products [35, 56, 71]. However, Nuijten et al. made an attempt to develop a standardized methodology for applying the DCF specifically to determine a drug price [51–55], which has already been applied in practice for the gene therapy product Zolgensma [73]. In this application of the DCF for drugs, as described by Nuijten & Vis a company’s cash flow is determined by starting with the profit before interest and tax (EBIT), adjusting for corporation tax, and obtaining the net operational result [51]. This is further adjusted by adding back depreciation, changes in working capital, and provisions, while subtracting investments and adding divestments. The net operational result is directly influenced by sales revenues, determined by quantity sold (Q) and price per unit (P). Q is a function of drug use per patient and the number of patients receiving the medicine, influenced by the conditions incidence, prevalence, probability of contraindications, and the market share of the product. Finally, in a more recent adaptation of the model, cost savings due to the substitution effect—wherein the introduction of a new medicine leads to reduced costs in other areas of healthcare spending—and the monetary gain expressed in QALYs —using the value of cost-effectiveness thresholds—are also incorporated [53]. This leads to the inclusion of a value-based element in the model. By considering all these factors, this method can be used to determine the expected future cash flows of a pharmaceutical company bringing a drug to the market, and ultimately determine its price.

The model, as outlined by Nuijten for drug pricing, operates under specific assumptions [51]: The cash flows considered in the model are limited from the time of patent registration and extend until the end of the patent period. Consequently, this analysis excludes any development or other costs incurred before obtaining the patent in year 1, treating them as sunk costs. The development costs are expressed as fixed monetary values, while the combined production and marketing costs are assumed to be 40% of the generated revenues. The cost of capital is approximated at 9% for pharmaceuticals, 11% for device firms, and 12% for biotech companies.

### Real-option rate of return pricing

Building upon the Gupta strategists’ ‘Cost of Opportunity’ report, which calculates the total R&D costs per new molecular entity (NME) [74], the Dutch "Fair Medicine" foundation has developed a pricing method that aims to comprehensively consider all costs associated with R&D upfront, including potential failure scenarios [57]. The model calculates the pppy, taking into account R&D costs, the average number of patients per year, patent period, production costs per patient per year, and a predefined profit percentage, as follows:

$$Pppy = \frac{{R\& D\,costs}}{{Average\,\# \,of\,ppy \times Patent\,period}} + Production\,costs\,ppy) \times \left( {1 + profit\% } \right)$$

The model aims to take into account all potential outcomes and relevant costs in medicine development, encompassing both out-of-pocket costs and the CoC. Here, it is acknowledged that not all drug development projects are successful. Consequently, failure costs are incorporated by multiplying the out-of-pocket costs per phase by the reciprocal of the cumulative success rate per phase, making it possible to estimate the cost per successful phase. Furthermore, by incorporating the CoC, the approach aims to ensure that the capital employed in R&D is evaluated not only based on its direct costs but also on the potential returns it could generate if invested elsewhere. To address global economic variations, the model employs worldwide differential pricing, adjusting prices based on GDP and public funding of countries or regions. Consequently, R&D costs are calculated as follows:

$$R\,\& \,D\,costs = (Out\,of\,pocket\,Costs + Failure\, costs + Costs\,of\,capital)$$

$$Failure\,costs\,=\,(Oop\, costs\, per\, phase)\times (1/cumulative\, success\, rate\, per\, phase)$$

$$Costs\, of\, capital\,=\, (R\&D\, costs\times (1+WACC\%{)}^{Average\, time\, till\, approval})\times (1-GDP\, deflator{)}^{Average\, time}$$

where,

- **OoP:** Out-of-Pocket,
- **WACC%:** Weighted average cost of capital %,
- **GDP:** Gross Domestic Product

In this regard, it should be noted that the model does not specify how to obtain the values of these input parameters, and whether real data or lump sums should be used. This would complicate the applicability of the model in practice. Furthermore, the same critique as to the cancer-drug pricing model applies that patent holders could potentially manipulate the patent years remaining to optimize their financial gains.

### Similarities & Differences between the models

There are clear differences, as well as similarities, among the drug pricing models identified in this study (Table 3). For example, it’s evident that many models incorporate both cost- and value-based elements, and the calculations often exhibit similarities (e.g., in both the cancer-drug pricing and AIM model, as well as the real-option rate of return method, R&D costs are divided by the number of eligible patients). However, there are clear differences in the assumptions of each model and how they are implemented. For instance, regarding the value-based aspect, ICER and Nuijtens DCF stand out as the only models that incorporate a direct value-driven parameter (QALY) into the calculation. In the AIM model, the value-based component is integrated as part of the innovation bonus provided, while the cancer-drug pricing model adds a profit margin based on clinical benefit. Similarly, examining the implementation of cost-based parameters reveals significant differences between the models. The AIM and cancer-drug pricing models, along with the real-option rate of return pricing method, consider both production and R&D costs. In contrast, Nuijtens DCF does not account for development or other costs incurred before obtaining the patent. Furthermore, while the AIM model and Nuijtens DCF work with lump sums, the other models rely on actual costs incurred (where feasible). The DCF and real-option rate of return method explicitly integrate the CoC required for drug development, making them the only models that consider the opportunity cost of capital in pharmaceutical development. What is further interesting to note is that both the cancer-drug pricing model and the real-option rate of return method incorporate the possibility of worldwide differential pricing, making them potentially more widely applicable across different nations, each with distinct economic contexts.

**Table 3:** Overview of identified drug pricing models

Model: Elements:	Value-based	Cost-based		Hybrid		
	ICER	Basic cost-based pricing	Real-option RoR	Cancer-drug pricing	AIM	(Nuijtens) DCF
**Cost-based**		**✔**	**✔**	**✔**	**✔**^**1)**^	**✔**^**2)**^
*R&D costs*			**✔**	**✔**	**✔**	**✔**^**1)**^
*Manufacturing costs*		**✔**	**✔**	**✔**	**✔**^**3)**^	**✔**^**4)**^
*Sales & marketing*		**✔**		**✔**^**5)**^	**✔**^**6)**^	**✔**^**4)**^
*Costs of failure*			**✔**			
*Costs of capital*			**✔**		^**7)**^	**✔**
**Value-based**	**✔**			**✔**	**✔**^**8)**^	**✔**
**Eligible patient pool:**			**✔**	**✔**	**✔**	**✔**
*Treatment rate*					**✔**	**✔**
*Market share*					**✔**	**✔**
**Patent period:**			**✔**	**✔**	**✔**	**✔**
**Innovation bonus:**					**✔**	
**Periodic price reassessment:**				**✔**		
**Worldwide differential pricing:**			**✔**	**✔**	^**9)**^	

1) Using lump sums

2) Does not include any incurred costs before obtaining the patent

3) Multiplied by 5 for orphan drugs

4) The sum of production and marketing costs are based on 40% of revenues

5) Capped at 30% of manufacturing costs

6) Capped at 20% of R&D costs

7) Ambiguous, as in some literature, upon which AIM bases its range for R&D expenses, the CoC are included

8) Incorporated in the innovation bonus

9) The model’s assumptions are tailored to the European market

Although all models, except for ICER calculation and basic cost-based pricing, factor in the number of eligible patients and market size in pricing calculations, only the AIM model and Nuijtens DCF include treatment rates and market shares as additional components. This potentially provides a more precise estimate of the number of patients receiving treatment. Additionally, it’s noteworthy that, excluding the ICER calculation and the cost-based pricing framework, all models take into account the patent period of the drug. Finally, only the cancer-drug pricing model incorporates a periodic reassessment of the price, where it is recalculated after three years, while the AIM and cancer drug pricing model, as well as Nuijtens DCF, consider (and cap) the costs of sales and marketing.

## Discussion

Given the complexity of drug development, one pricing model may not be suitable for all pharmaceutical products. The models identified in this study each have distinct attributes that render them more or less suitable for specific indications or types of products. Here, it should be noted that basic cost-based (plus) pricing—only looking at manufacturing costs—would only be suitable for pricing generic medicines, which have a low development risk profile. Conversely, basic cost-plus pricing is unlikely to offer adequate incentives for pharmaceutical innovation, such as novel pathways or targets.

To calculate the costs of *common drugs* that are frequently prescribed with no innovative mechanism of action or uncertainty about the effectiveness, ICER calculations seem particularly suitable. As these take into account the cost-effectiveness of the current treatment option, they would allow for a comprehensive evaluation of the value proposition of new drugs compared to established treatments, ensuring a thorough assessment of their economic and clinical impact. Consequently, healthcare decision-makers can make informed choices that prioritize the more efficient and equitable allocation of healthcare resources to maximize health gains. However, with an explicit ICER threshold, there is a risk that pharmaceutical companies may set the price of their product to match this maximum specified threshold, potentially pushing health systems to consistently pay the maximum amount they are willing to pay [75]. This aligns with perhaps the most significant drawback of ICER calculations, where the absence of cost-based elements and incurred costs of drug development restrict transparency in drug pricing, thereby fuelling the ongoing debate regarding whether drug prices are excessively high. Conversely, models integrating cost-based elements could provide a more precise representation of the investments incurred, although the challenge here is to achieve transparency and gaining insight into the actual incurred costs. Additionally, it is important to recognize the subjective and context-dependent nature of value in pharmaceutical pricing, which complicates VBP and the use of ICER calculations. The perceived value of a drug can vary significantly depending on the healthcare context, including the specific needs of patients, the availability of alternative treatments, and regional economic conditions. For instance, what is considered a high-value drug in one country or healthcare system may not be perceived the same way in another due to differences in healthcare priorities, economic resources, and patient needs.

For *precision medicine* and drugs targeting small patient populations, the hybrid models and the real-option rate of return method, stand out. These pricing models take into account the number of eligible patients. In situations where the patient pool is limited, these models allow for a higher price to be set, providing an incentive for pharmaceutical developers to invest in the development of treatments for diseases that might otherwise be overlooked. This would also align with the industry’s shift towards niche markets. It’s worth mentioning that the AIM model and Nuijtens DCF method include treatment rates and market shares, offering a more precise estimation of the patient population undergoing treatment. Additionally, the AIM model considers production costs, scaling them by a factor of five for orphan drugs to address the challenges of limited production volumes and compensate for the lack of economies of scale.

For highly *innovative drugs*, including orphan drugs, these models also seem fitting, given the uncertainty surrounding the long-term effectiveness of these products, which complicates VBP [24–26]. Nuijtens DCF and the real-option rate of return method stand out in this context as they explicitly consider the CoC, which tends to be higher for riskier ventures, such as innovative drugs, making them an important factor to consider [76]. However, it is a political question to what extent health payers should accommodate these CoC and the return requirements of investors. Moreover, these models do not possess an innovation bonus like AIM does, incentivizing the development of drugs with a novel mechanism of action. Meanwhile, the cancer-drug pricing model incorporates periodic reassessment, allowing for adjustments to the set price based on new information, changes in indications, or emerging clinical benefits. This is crucial in rapidly evolving fields such as oncology and gene therapy.

Finally, for *repurposed drugs*, models with cost-based elements seem more suitable than VBP. When focusing solely on the value that a medicine brings, there is a risk of inflated prices that are disproportionate to the investments made [38, 77]. On the other hand, models with cost-based elements should take into account the costs incurred to bring a medicine to market. Because these investments are expected to be lower for repurposed medicines, these models could potentially result in a price that aligns more appropriately with the incurred investments. It should be noted that full cost-based pricing, where only production costs are considered, would not provide an adequate incentive for repurposing projects, as there are still investments required for repurposing studies [78]. Notably, the AIM model seems particularly well-suited for repurposed medicines, as evidenced by its innovation bonus calculation, which considers additional indications, and the fact that it reduces R&D costs for repurposed products.

It is imperative to recognize that there is *no ‘one-size-fits-all’ approach* to drug pricing. Selecting an appropriate pricing strategy depends on specific characteristics inherent to the drug, the therapeutic area it addresses, and the availability of alternative treatments. Additionally, it is important to consider the applicability of pricing models across different economic contexts, taking into account diverse GDPs and market conditions. Consequently, rather than focusing on applying one particular pricing model, the emphasis should be on identifying which components of which model are relevant for pricing a new product. A policy recommendation would therefore be to work towards an integrated *differential drug pricing framework* that accommodates the diverse landscape of pharmaceutical pricing. This framework should encourage flexibility in pricing strategies, allowing for the adaptation of components within this model based on the unique attributes of the drug in question. The price resulting from this framework could serve as a benchmark for price negotiations between health payers and pharmaceutical companies. By offering transparency in the incurred costs and substantiating why certain components should or should not be considered, companies could then request an adjusted price. Conversely, health payers could also require certain justifications from pharmaceutical companies when pricing a product. Finally, as more becomes known about the long-term effectiveness of a product, the price could be adjusted accordingly.

The implementation of such a framework, would provide health payers with leverage in price negotiations, which is lacking when solely relying on CEAs and ICER’s. Future research could delve into how such a differential pricing framework might take shape, including an exploration of the necessary infrastructure and policies required, adding an additional layer of interest to the inquiry. This avenue of research would provide valuable insights, contributing to improved capabilities to determine whether something is an affordable price.

It should be stressed however, that without accurate data about the incurred costs, even an integrated pricing model can’t resolve the challenges of pricing new medications. Before discussing which elements should or should not be considered and determining what a ‘fair’ price might be, health payers need insight into actual costs. This highlights the critical need for transparency in the pharmaceutical industry, a point emphasized by governments, stakeholders, and academics [79–82]. Following up on that, there’s a risk of companies exaggerating costs to secure higher prices, highlighting the importance of *independent audits*. These audits would help verify the authenticity and accuracy of disclosed costs, offering an additional layer of assurance and promoting trust in the pricing negotiation process.

This study has several limitations that warrant consideration. One potential limitation is the reliance on English and Dutch language publications, which may introduce bias by excluding valuable insights from non-English literature. For instance, there were publications describing cost-based pricing strategies in Japan, which, due to being in Japanese, could not be included for review. Furthermore, this study exclusively examines pricing models with tangible input variables and does not delve deeper into the policy aspects of drug pricing and accessibility nor into pricing schemes used to manage controlled access to medicines, potentially omitting interesting insights from existing approaches. During the literature review, numerous approaches were identified that were not addressed due to a lack of a concrete model to determine the price of a medicine. Examples of these include delinkage approaches, the diagnosis-confirmation model, indication- and outcome-specific pricing models, and the milestone-based performance-based pricing model [83–87]. The performance and outcome-based models, in particular, could provide an extra dimension for dynamic pricing, where, for example, components within the advocated differential drug pricing model can be adjusted as there is more evidence of effectiveness. However, even within these schemes, a model is required to establish an initial price that can be used as a basis for further adjustments. This study provides insights into constructing and applying these models, laying the groundwork for refining such schemes to manage controlled access to medicines.

## Figures and Tables

**Appendix 1:**
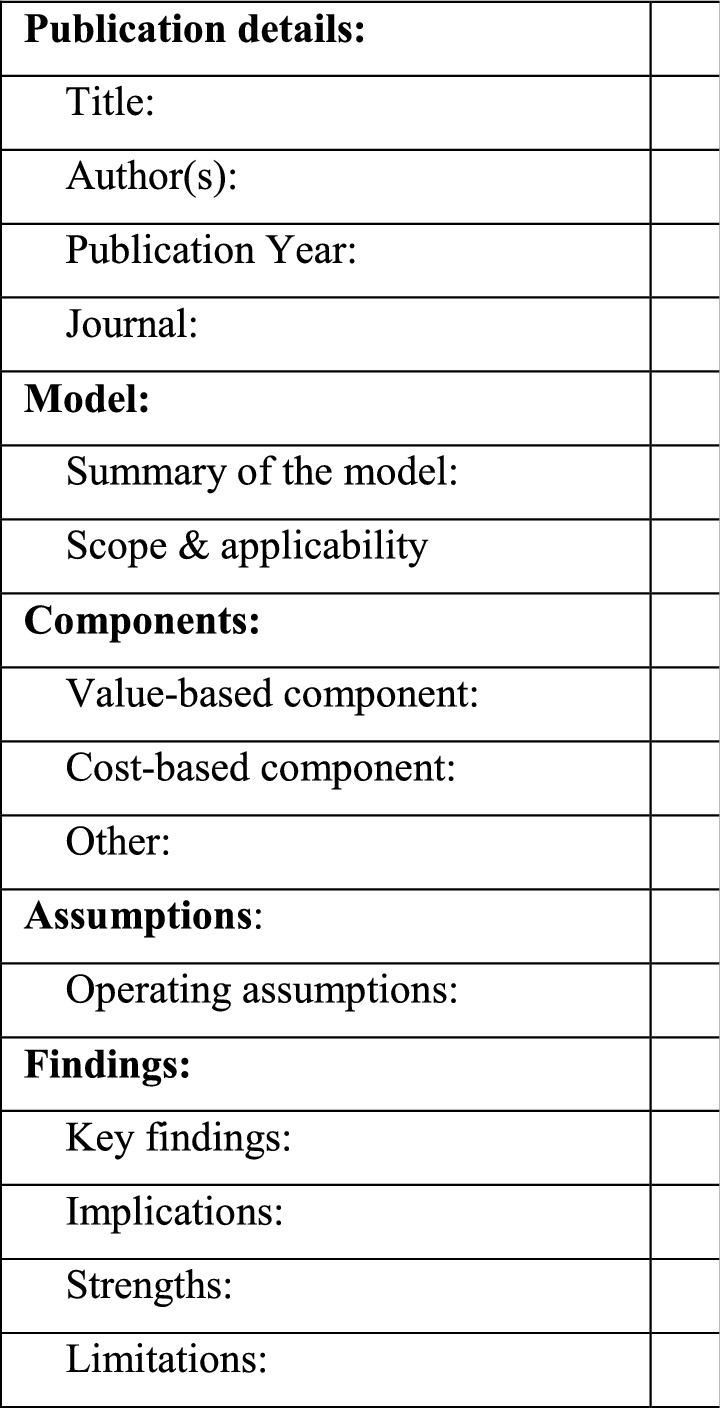
Data extraction form

## Abbreviations

AIM: The International Association of Mutual Benefit Societies
CEA: Cost-effectiveness analysis
CoC: Costs of capital
DCF: Discounted cash flow
GDP: Gross domestic products
HTA: Health technology assessment
ICER: Incremental cost-effectiveness ratio
M&A: Merger and acquisition
NME: New medical entity
OECD: Organisation for Economic Co-operation and Development
Pppy: Price per patient per year
QALY: Quality -adjusted life year
R&D: Research and development
VBP: Value based pricing
WHO: World health organisation
